# Supplementary material for: Nutritional Quality of Gluten-Free Bakery Products Labeled Ketogenic and/or Low-Carb Sold in the Global Market
Source: Foods. 2022 Dec 18;11(24):4095. doi: 10.3390/foods11244095 (PMC9778343; doi:10.3390/foods11244095)
Supplement: Supplementary file 1 [file foods-11-04095-s001.zip › foods-2003482-supplementary.pdf]

Table S1: List of ingredients of baking flour mixes

Ingredients	K-LC	K	LC	STD
**Flours**	Almond flour coconut flour cassava flour lupin flour brown rice, linseed flour chickpea flour	Almond flour, coconut flour, cocoa powder, linseed flour, tiger nut flour, cassava flour chickpea flour, brown rice flour lupin flour, rice flour	Rice flour cocoa powder corn flours cassava flour brown rice flour almond flour oat flour almond soybean flour coconut flour, millet flour, coconut flour, chickpea flour, sorghum flour, navy bean flour, lupin flour	Cassava flour almond flour coconut flour cocoa powder brown rice flour amaranth flour white rice flour oat flour chickpea flour corn flours sorghum flour potato flour rice flour
**Fibers**	Xanthan gum psyllium seed husks soluble corn fibre, inulin, cassava fibre oat fibre sugar cane fiber resistant dextrin guar gum pea fibre apple fiber, cellulose	Xanthan gum inulin psyllium seed husks cassava fibre soluble corn fibre oat fibre sugar cane fiber guar gum<br>Resistant dextrin	Psyllium seed husks xanthan gum guar gum inulin citrus fibre, resistant starch, oat fiber, pea fiber	Guar gum psyllium seed husks inulin cassava fibre carrageenan
**Sugars**	-	Cane sugar, oligosaccharides	White sugar cane sugar, brown sugar, glucose, resistant maltodextrin, polydextrose	Cane sugar, coconut palm sugar brown sugar glucose maltodextrin
**Starches**	Cassava starch corn starch pea starch	Cassava starch corn starch potato starch	Corn starch potato starch cassava starch rice starch	Potato starch modified corn starch modified cassava starch
**Fats**	Coconut oil, sunflower oil, palm oil	Cocoa fat, sunflower seed oil - high oleic acid coconut oil palm oil<br>Low erucic acid rapeseed oil, skimmed milk powder	Sunflower seed oil palm oil coconut oil butter sunflower seed oil safflower seed oil, cocoa fat	Palm oil palm kernel oil skimmed milk powder mono- and di-glycerides of fatty acids low erucic acid rapeseed oil milk fats
**Proteins**	Dried eggs, pea protein, whey protein concentrates, milk protein isolate rice protein	Eggs whey protein concentrate pea protein<br>Egg whites	Eggs, pea protein, whey protein, cereal gluten	Egg whites
**Sweeteners**	Erythritol xylitol allulose steviol glycoside, stevia, maltitol, sucralose	Erythritol stevia steviol glycoside extract xylitol allulose	Erythritol, stevia, xylitol, allulose, sucralose, steviol glycoside	Allulose
**Sodium**		Sodium hydrogen carbonate, salt	Sea salt sodium hydrogen carbonate	Iodized salt

Table S2: List of ingredients of bread products

Ingredients	K-LC	K	LC	STD
**Flours**	Almond flour coconut flour konjac flour cassava flour hazelnut flour coconut palm sugar	Coconut flour almond flour linseed chia seed cassava flour arrowroot flour konjac flour almond	Coconut flour almond flour cornflours cassava flour soybean amaranth sweet potato flour	Rice flour cocoa powder cornflours cassava flour brown rice flour almond flour oat flour almond quinoa soybean flour coconut flour, millet flour brown rice flour soybean flour
**Fibers**	Psyllium seed husks, inulin, bamboo fibre guar gum rice bran wheat fibre citrus fibre, cellulose	Psyllium seed husks xanthan gum guar gum inulin carob bean gum bamboo fibre rice bran	Psyllium seed husks xanthan gum inulin powdered cellulose bamboo fibre rice bran oat fibre	Psyllium seed husks xanthan gum guar gum inulin citrus fibre
**Sugars**		Maple syrup	Glycerol	White sugar cane sugar, brown sugar, glucose glycerol rice syrup agave syrup
**Starches**	Cassava starch, resistant tapioca starch corn starch	Corn starch modified cassava starch (food) resistant tapioca starch potato starch	Potato starch cassava starch resistant tapioca starch corn starch	Corn starch potato starch cassava starch rice starch
**Fats**	Coconut oil, mozzarella cheese, sunflower seed oil olive oil sunflower fat	Coconut oil sunflower seed oil coconut oil avocado oil cream cheese almond butter, cream coconut mozzarella cheese milk poppy seed palm oil sesame seed coconut cream olive oil cheddar cheese butter ghee	Coconut oil mozzarella cheese full fat milk sunflower seed olive oil skim milk almond butter	Sunflower seed oil palm oil coconut oil butter sunflower seed oil milk rapeseed oil palm fat mono- and di-glycerides of fatty acids black sesame seed, margarine, cheese and cheese products linseed chia seed, olive oil full fat milk safflower seed oil,
**Proteins**	Egg whites, pumpkin seed protein hemp protein	Egg whites eggs pumpkin seed protein hemp protein soybean proteins	Egg whites isolated soy protein pumpkin seed protein	Eggs, soybean protein, pea protein
**Sweeteners**				
**Sodium**	Sodium hydrogen carbonate	Sodium hydrogen carbonate	Sodium hydrogen carbonate sea salt	Sea salt sodium hydrogen carbonate

Table S3: List of ingredients of cakes, pastries, and sweet goods

Ingredients	K-LC	K	LC	STD
**Flours**	Almond flour coconut flour cassava flour lupin flour brown rice, linseed flour, oat flour lupin flour	Almond flour coconut flour lupin flour, peanut flour, chia flour cassava flour cocoa powder linseed flour, cashew nut flour, arrowroot flour (food), soybean flour sunseed flour	Almond flour soybean flour coconut flour	Rice flour cocoa powder corn flours cassava flour brown rice flour almond flour oat flour almond soybean flour coconut flour, millet flour brown rice flour cocoa powder sorghum flour konjac flour bean flour
**Fibers**	Xanthan gum psyllium seed husks soluble corn fibre, inulin, cassava fibre oat fibre sugar cane fiber guar gum powdered cellulose bamboo fibre	Xanthan gum inulin gum arabic, guar gum soluble corn fibre, cassava fibre, psyllium seed husks, powdered cellulose, bamboo fibre	Xanthan gum soluble corn fibre powdered cellulose inulin, guar gum bamboo fibre, psyllium seed husks	Psyllium seed husks xanthan gum guar gum inulin citrus fibre
**Sugars**	Corn maltodextrin glycerol glucose	Glycerol polydextrose cocoa liquor, oligosaccharides white sugar glucose, date, coconut palm sugar, glycerol,	Glycerol	White sugar cane sugar, brown sugar, glucose glycerol rice syrup agave syrup invert sugars maltodextrin
**Starches**	Cassava starch corn starch pea starch potato starch rice starch	Cassava starch corn starch	Cassava starch	Corn starch potato starch cassava starch rice starch
**Fats**	Coconut oil, sunflower oil, palm oil, olive oil, milk, rice bran oil, butter, coconut cream, full fat milk, butter cocoa fat, palm oil, peanut butter, cream coconut milk butter hazelnut butter	Coconut oil butter cocoa fat palm oil almond butter, cream, peanut butter full fat milk, medium chain triglycerides, milk palm kernel oil avocado oil butter ghee egg yolks, sunflower oil salted butter almond butter skim milk peanut butter	Palm kernel oil coconut oil palm oil cocoa fat sunflower seed oil, butter, peanut butter, olive oil, milk rapeseed oil	Sunflower seed oil palm oil coconut oil butter sunflower seed oil milk rapeseed oil palm fat mono- and di-glycerides of fatty acids black sesame seed, margarine, cheese and cheese products linseed chia seed, olive oil full fat milk safflower seed oil, cocoa fat butter milk sweetened condensed milk butter fat
**Proteins**	Eggs isolated soy protein casein milk protein concentrate	Eggs egg whites, pea protein hydrolyzed collagen dried eggs, casein soy protein concentrates	Eggs whey protein isolate hydrolyzed collagen casein, milk protein	Eggs, spirulina extract
**Sweeteners**	Erythritol, xylitol allulose steviol glycoside, stevia, maltitol, sucralose	Erythritol stevia extract steviol glycoside allulose xylitol stevioside sucralose	Erythritol stevia sucralose, allulose, xylitol	Sorbitol
**Sodium**	Rock salt sodium hydrogen carbonate	Sodium hydrogen carbonate salt	Sodium hydrogen carbonate	Sea salt sodium hydrogen carbonate

Table S4: List of ingredients of savory biscuits and crackers

Ingredients	K-LC	K	LC	STD
**Flours**	Almond flour coconut flour cassava flour lupin flour brown rice, linseed flour chickpea flour, soybean flour	Coconut flour almond flour linseed chia seed cassava flour arrowroot flour konjac flour almond, soy flour, lupin flour, brown rice flour	Almond coconut flour brown rice flour linseed flour rice flour soybean flour oat flour cassava flour chickpea flour lupin flour quinoa flour	Rice flour cocoa powder cornflours cassava flour brown rice flour almond flour oat flour almond quinoa soybean flour arrowroot flour buckwheat flour coconut flour, millet flour
**Fibers**	Xanthan gum psyllium seed husks soluble corn fibre, inulin, cassava fibre oat fibre sugar cane fiber resistant dextrin guar gum pea fibre apple fiber, cellulose	Psyllium seed husks xanthan gum guar gum inulin carob bean gum bamboo fibre rice bran	Xanthan gum inulin guar gum cassava fibre	Xanthan gum guar gum inulin
**Sugars**	Corn maltodextrin	Maple syrup	White sugar polydextrose fructo-oligosaccharides	White sugar cane sugar m coconut palm sugar brown sugar
**Starches**	Cassava starch corn starch pea starch potato starch	Corn starch modified cassava starch (food) resistant tapioca starch potato starch, cassava starch potato starch	Corn starch cassava starch resistant tapioca starch potato starch	Corn starch potato starch cassava starch rice starch
**Fats**	Coconut oil, sunflower oil, palm oil, olive oil, milk, rice bran oil, butter, coconut cream, cheese, full fat milk	Coconut oil sunflower seed oil coconut oil avocado oil cream cheese almond butter, cream coconut mozzarella cheese milk poppy seed palm oil sesame seed coconut cream olive oil cheddar cheese butter ghee, rice bran oil, milk, butter, coconut cream butter milk	Butter cocoa fat coconut oil palm oil milk mono- and di-glycerides of fatty acids, butter palm kernel oil skimmed milk powder soybean oil milk solids rice bran oil butter milk cream cheese	Cocoa fat sunflower seed oil palm oil coconut oil butter sunflower seed oil milk rapeseed oil palm fat mono- and di-glycerides of fatty acids skimmed milk powder, black sesame seed, margarine, cheese and cheese products linseed chia seed poppy seed pumpkin seed olive oil
**Proteins**	Dried eggs whey protein concentrate milk protein, hemp protein, algae	Egg whites eggs pumpkin seed protein hemp protein soybean proteins, algae	Eggs egg soy protein isolates whey protein concentrate	Eggs
**Sweeteners**	Erythritol xylitol allulose steviol glycoside, stevia, maltitol, sucralose	Annatto stevia extract		
**Sodium**	Sodium hydrogen carbonate salt	Sodium hydrogen carbonate	Sodium hydrogen carbonate salt	Sea salt sodium hydrogen carbonate

Table S5: List of ingredients of sweet biscuits and cookies

Ingredients	K-LC	K	LC	STD
**Flours**	Almond flour coconut flour cassava flour lupin flour brown rice, linseed flour , oat flour	Almond flour coconut flour lupin flour, peanut flour, chia flour cassava flour cocoa powder linseed flour,, cashew nut flour, arrowroot flour (food)	Almond coconut flour brown rice flour linseed flour rice flour soybean flour oat flour cassava flour	Rice flour cocoa powder cornflours cassava flour brown rice flour almond flour oat flour almond quinoa soybean flour arrowroot flour buckwheat flour coconut flour
**Fibers**	Xanthan gum psyllium seed husks soluble corn fibre, inulin, cassava fibre oat fibre sugar cane fiber guar gum	Xanthan gum inulin gum arabic, guar gum soluble corn fibre, cassava fibre, psyllium seed husks	Xanthan gum inulin guar gum cassava fibre psyllium seed husks	Xanthan gum guar gum inulin
**Sugars**	Corn maltodextrin glycerol glucose	Glycerol polydextrose cocoa liquor, oligosaccharides white sugar glucose, date, coconut palm sugar	Glycerol white sugar polydextrose fructo-oligosaccharides	White sugar cane sugar maltodextrin coconut palm sugar brown sugar glucose syrup tapioca syrup polydextrose caramel i plain
**Starches**	Cassava starch corn starch pea starch potato starch	Cassava starch	Corn starch cassava starch	Corn starch potato starch cassava starch rice starch
**Fats**	Coconut oil, sunflower oil, palm oil, olive oil, milk, rice bran oil, butter, coconut cream, full fat milk, butter cocoa fat, palm oil, peanut butter, cream	Coconut oil butter cocoa fat palm oil almond butter, cream, peanut butter full fat milk, medium chain triglycerides, milk palm kernel oil avocado oil butter ghee egg yolks, sunflower oil salted butter	Butter cocoa fat coconut oil palm oil milk mono- and di-glycerides of fatty acids peanut butter palm kernel oil skimmed milk powder soybean oil milk solids margarine, cream	Cocoa fat sunflower seed oil palm oil coconut oil butter sunflower seed oil milk rapeseed oil palm fat mono- and di-glycerides of fatty acids skimmed milk powder
**Proteins**	Eggs isolated soy protein	Eggs egg whites, pea protein hydrolyzed collagen dried eggs	Eggs egg whites isolated soy protein whey protein concentrate milk proteins whey protein isolate	Eggs
**Sweeteners**	Erythritol xylitol allulose steviol glycoside, stevia, maltitol, sucralose	Erythritol stevia extract steviol glycoside allulose xylitol stevioside sucralose	Erythritol stevia allulose sucralosemaltitol xylitol steviol glycoside sorbitol	Sorbitols sucralose erythritol stevia
**Sodium**	Sodium hydrogen carbonate salt	Sodium hydrogen carbonate salt	Sodium hydrogen carbonate salt	Sea salt sodium hydrogen carbonate
